# Ni-Doped ZnS Nanospheres Decorated with Au Nanoparticles for Highly Improved Gas Sensor Performance

**DOI:** 10.3390/s18092882

**Published:** 2018-08-31

**Authors:** Furu Zhong, Zhaofeng Wu, Jixi Guo, Dianzeng Jia

**Affiliations:** Key Laboratory of Energy Materials Chemistry, Ministry of Education, Key Laboratory of Advanced Functional Materials, Autonomous Region, Institute of Applied Chemistry, Xinjiang University, Urumqi 830046, China; zhfuru@shzu.edu.cn (F.Z.); wzf911@mail.ustc.edu.cn (Z.W.); jxguo1012@163.com (J.G.)

**Keywords:** ZnS nanosphere, Au nanoparticle, room temperature, formaldehyde sensor

## Abstract

Novel Ni-doped wurtzite ZnS nanospheres decorated with Au nanoparticles (Au NPs–ZnS NSs) have been successfully fabricated using a simple method involving vacuum evaporation followed by an annealing process. This transition metal-doped gas sensor had high responsivity, extremely fast response and recovery time, and excellent selectivity to formaldehyde at room temperature. The response and recovery time are only 29 s and 2 s, respectively. Since ZnS is transformed into ZnO at a high temperature, superior room temperature-sensing performance can improve the stability and service life of the sensor. The improvement in sensing performance could be attributed to the reduced charge-transfer distance resulting from the creation of a local charge reservoir layer, and the catalytic and spillover effect of Au nanoparticles. The rough and porous spherical structure can also facilitate the detection and diffusion of gases. The as-prepared Au NPs–ZnS NSs are considered to be an extremely promising candidate material for gas sensors, and are expected to have other potential applications in the future.

## 1. Introduction

Formaldehyde is a gas that is harmful to human health. The World Health Organization (WHO) states that exposure to formaldehyde for 30 min in an environment with a concentration greater than 0.08 ppm will harm human health [1]. However, formaldehyde is widely found in building materials, such as chemical adhesives. It has also become one of the main pollutant gases in the indoor environment. A previous study has shown that the formaldehyde concentration in the exhaled gas of lung cancer patients reaches 83 ppb, while it is 48 ppb for healthy counterparts [2]. Therefore, it is highly desirable to monitor the environmental formaldehyde concentration in real time by using high-precision and high-sensitivity portable gas sensors. 

Zinc sulfide (ZnS), which is a II–VI semiconducting material with a wide band gap (3.80 eV), has attracted the attention of many researchers because it can be used in field emitters [3], lasers [4], phosphors [5], solar cells [6], gas sensors [7], and so on. Since ZnS has a crystal structure similar to that of ZnO, rich trapped surface states, and sulfur vacancies, ZnS gas sensors have been widely used in the detection of trace analytes, such as ethanol [8], formaldehyde [9], and NO_2_ [10]. However, most ZnS chemiresistor gas sensors have a low response and low sensitivity at room temperature, such as 32% toward 5 ppm formaldehyde [9], which is much less than the sensitivity required to detect concentrations that are harmful to human health according to WHO. A higher working temperature can improve the performance of sensors, but ZnS easily oxidizes to ZnO at a higher temperature, reducing the performance of the sensor. Therefore, it is important for a gas sensor to operate at room temperature. Nanocrystallization and doping are two important preparation methods that can improve the detection performance of gas sensors. Many studies have been reported in this field [11,12,13,14,15], and gas sensors have been reported to work at room temperature and detect some gases with good selectivity. However, most of the previously reported room temperature gas sensors use ZnO nanomaterial, and few articles in the literature have reported room temperature gas sensors based on ZnS nanomaterial. Other studies have shown that sulfide gas sensors have high selectivity and sensitivity [16,17,18]. Nevertheless, these previous works mainly reported gas sensing at elevated temperatures. Moreover, ZnS is considered as a substitute for CdS because of its simple preparation method, low cost, and low toxicity [19]. Compared with ZnO, ZnS has a wider forbidden band and better thermal stability [20].

Some studies have shown that surface defect states on semiconductors play a very important role in exchanging the chemisorption of target gas molecules [21]. Therefore, the increase of the surface defect states of ZnS nanomaterial may significantly increase the response of the sensor. On the other hand, the response speed of existing formaldehyde gas sensors is far less than that required for practical applications. For example, a previously reported formaldehyde gas sensor shows a response time of 100 s and recovery time of 88 s to 60 ppb [22]. Thus, it is important to develop novel methods to reduce the response/recovery time. As we know, the charge-exchange rate between the sensing materials and the absorbed target molecules determines the response time of the sensor [23]. By introducing an impurity energy band state, transition metal doping can significantly improve the mobility of charge carriers. Studies have shown that surface functionalization with noble metals is a simple and effective method to enhance the sensing properties, such as response, response/recovery speed, and reproducibility, at room temperature [24,25]. However, thus far, no study has revealed the gas-sensitivity characteristics of transition metal-doped ZnS 0-dimensional (0 D) nanostructures functionalized with a noble metal catalyst, such as Au, despite decoration with Au nanoparticles being essential to ZnS gas sensors.

In this work, we combine the two methods of transition metal doping and Au decoration to remarkably improve the sensing performances of a formaldehyde gas sensor based on ZnS nanospheres at room temperature. The novel method of decoration with Au nanoparticles is simple and achieved by vacuum evaporation followed by an annealing process. Thus, this method is environmentally friendly and pollution-free, and it does not use any surfactants or toxic organic solvents. The sensing properties of ZnS nanospheres decorated with Au nanoparticles (Au NPs–ZnS NSs) were tested. An ultralow formaldehyde concentration of 67.5 ppb can be detected at room temperature with excellent selectivity. The as-prepared Au NPs–ZnS NSs are considered to be an extremely promising candidate material to realize portable, real-time, and cheap gas sensors, and they are expected to have other potential applications in the future. 

## 2. Materials and Methods

Ni-doped ZnS nanospheres were fabricated using the method reported in a previous work [26]. In brief, 100 mmol of Zn(CH3COOH)_2_·2H_2_O was first dissolved in 100 mL of deionized water by stirring. Subsequently, 10 mmol Ni(CH_3_COOH)_2_·4H_2_O was added with stirring, followed by ultrasonication for 10 min at room temperature. Then, 20 mL of an aqueous solution containing 100 mmol Na_2_S·9H_2_O was added dropwise into the reaction system, and the mixture was vigorously stirred for 12 h at room temperature. The resulting Ni-doped ZnS nanospheres were centrifuged and washed thoroughly with deionized water and absolute ethanol, and finally dried in an oven at 60 °C for 24 h.

In the second step, a gold thin film was optionally deposited on the surfaces of the as-synthesized Ni-doped ZnS nanospheres by using a DC sputtering technique (substrate temperature: room temperature, power: 20 mA, working pressure: 1.9 × 10^−2^ Torr, and process time: 100 s). The thickness of the gold thin film deposited on the nanospheres was approximately 10 nm; this gold would be used as a catalyst for the enhancement of sensing properties of the nanomaterial. Subsequently, the Au-coated nanospheres were annealed at 500 °C for 1 h in a muffle furnace. 

Finally, a 2 mg sample was added to 5 mL of deionized water and sonicated for 30 min to prepare colloid solution of nanospheres. These solutions were then dripped onto the Au interdigital electrode (IDE) patterned chip, and dried at room temperature. The space between the electrodes was fixed at 20 µm, and the two electrodes were connected by a channel of interconnected nanospheres. The IDE chip was mounted to sensing equipment made in-house, to examine the formaldehyde gas-sensing performance at room temperature when a potential difference of 1 V was applied between the two electrodes. The current, as well as response and recovery times, were measured when the variation in the current of the sensors reached 90% of the equilibrium value. That is, the response time was defined as the period from the time when the gas sensor came into contact with the gas to be detected to the time when the variation of current reached 90% of (I_g_–I_a_), and the recovery time is the period from the time when the gas sensor was separated from the gas to be detected to the time when the variation of current reached 90% of (I_g_–I_a_). The response (R) was defined as I_g_/I_a_, where I_a_ and I_g_ represent the current value when the sensors were exposed to air and the target gas, respectively. The structure of the sensing equipment used in this experiment is described elsewhere [22]. When testing various gases, the temperatures were controlled at room temperature (~300 K), and the air humidity was approximately 35%. 

Morphological and structural investigations of ZnS nanospheres were, respectively, carried out using a field emission scanning electron microscopy (FESEM, Hitachi S-4800, Hitachi, Japan) and a diffractometer (Bruker D8 Advance Diffraction) in the 2θ range from 20° to 80° with Cu Kα radiation (λ = 0.15405 nm) at 40 kV and 40 mA. The electrical signals of the sensor were characterized via a Zennium Pro electrochemical workstation (Zennium Pro, s-n40201, Zahner, Germany). 

## 3. Results and Discussion

### 3.1. Structure and Morphological Characteristics

**Figure 1:**
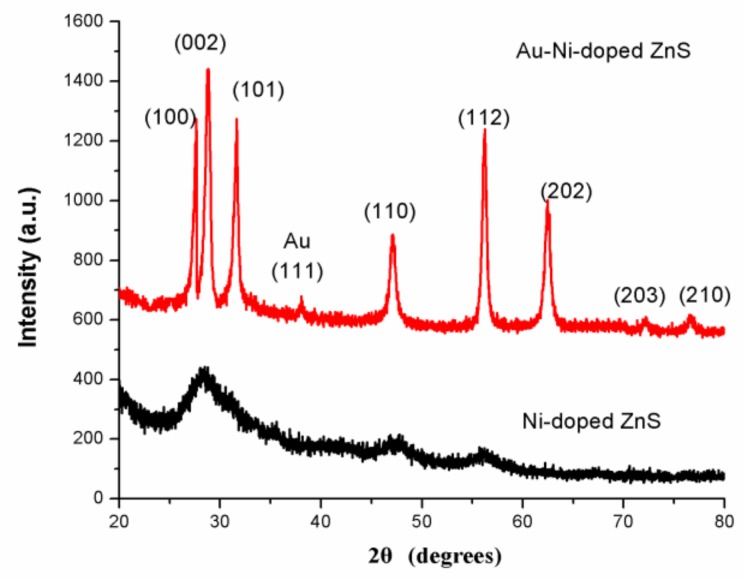
XRD pattern of Au–Ni-doped ZnS nanospheres (**up**) and Ni-doped ZnS nanospheres (**down**).

Figure 1 shows the X-ray diffraction (XRD) patterns of as-prepared Ni-doped ZnS nanospheres with and without Au decoration. There were no sharp reflection peaks in the XRD pattern of Ni-doped ZnS without Au decoration, indicating that the crystalline form of ZnS nanomaterial is not very good. However, in the samples with Au decoration after annealing, some sharp diffraction peaks corresponding to (100), (002), (110), (112), (202), and (203) planes are well matched with the standard hexagonal wurtzite phase of ZnS in JCPDS file No. 75-1547. The XRD patterns of Ni-doped ZnS nanospheres with Au decoration also revealed that Au nanoparticles were crystalline. 

**Figure 2:**
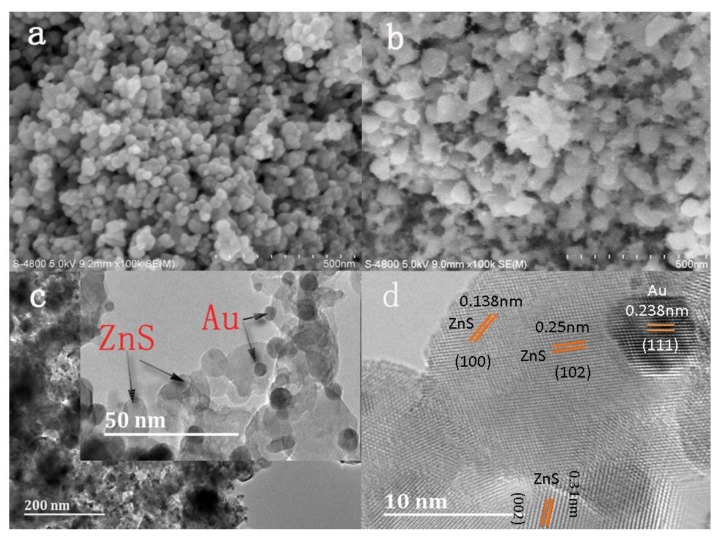
SEM image of (**a**) pristine ZnS nanosphere and (**b**) Au-decorated Ni-doped ZnS nanospheres after annealing. (**c**) TEM image and (**d**) HRTEM image of Au-decorated Ni-doped ZnS nanospheres.

The surface morphologies of Ni-doped ZnS nanospheres with and without Au decoration are shown in Figure 2a–d. Ni-doped ZnS nanospheres both with and without Au decoration consist of numerous nanospheres with a diameter approximately 40 nm. The morphology of nanospheres modified by Au nanoparticles did not change significantly after annealing, apart from becoming rougher. Numerous nanoparticles are accumulated at the surface, which may improve the sensing performance of the gas sensor. 

The obtained Au nanoparticle-decorated Ni-doped ZnS nanospheres were further characterized by TEM and high-resolution TEM (HRTEM). The Au metallic particles exhibit a dark contrast in the TEM image. Many small particles scattered on the surface of the nanospheres and partial Au nanoparticles were mounted on the surface layer of the nanospheres, as shown in Figure 2c. An HRTEM image of Au nanoparticle-decorated Ni-doped ZnS nanospheres is shown in Figure 2d. A clear lattice spacing of approximately 0.238 nm can be indexed to the (111) plane of Au (white), and interplanar distances of 0.31 nm, 0.25 nm, and 0.138 nm can be indexed to the (002), (102), and (100) planes of ZnS (black), respectively. 

The gold film deposited by DC sputtering consists of gold nanoparticles. Therefore, the melting point of gold nanoparticles is much lower. Under the condition of annealing at 500 °C in a nitrogen atmosphere, the gold film is heated and melted. On one hand, the gold melt has a tendency to wet the substrate, thereby wrapping onto the surface of ZnS nanospheres; on the other hand, the thin film on the substrate surface has a tendency to shrink into a spherical shape under the action of surface tension [27]. Thus, smaller gold nanoparticles were obtained. 

### 3.2. Gas-Sensing Properties

As we know, precious metals, such as Au, exhibit unexpected electronic and catalytic properties as the particle size approaches the nanoscale. We believe that Ni-doped ZnS nanospheres decorated with nanoscale Au particles will greatly improve the sensing performance. Here, we have fabricated an Au-decorated Ni-doped ZnS (Ni-doped ZnS-Au) gas sensor and investigated its sensing performance to formaldehyde at room temperature. 

**Figure 3:**
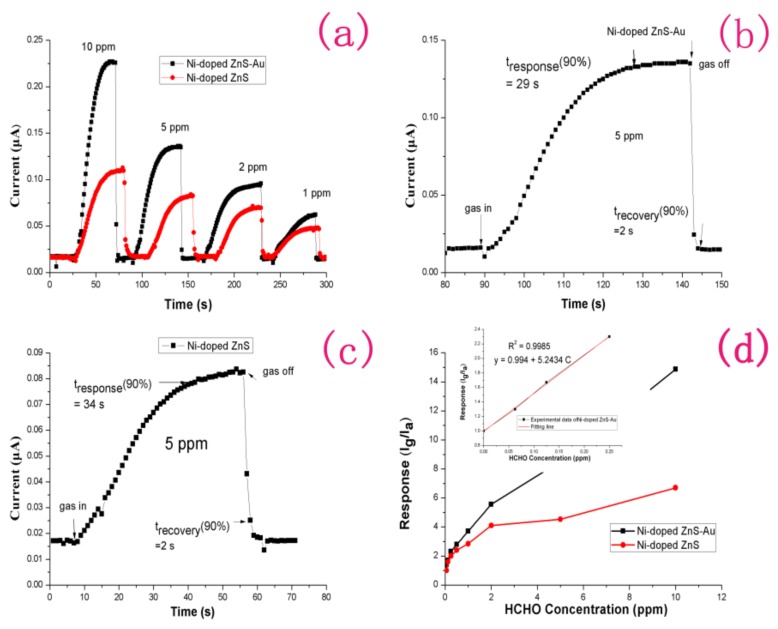
(**a**) Dynamic response of the gas sensors based on Ni-doped ZnS nanospheres and Au-decorated Ni-doped ZnS nanospheres at room temperature. (**b**) Enlarged part of (**a**) for the gas sensor based on Au-decorated Ni-doped ZnS nanospheres at 5 ppm HCHO. (**c**) Enlarged part of (**a**) for the gas sensor based on Ni-doped ZnS nanospheres at 5 ppm HCHO. (**d**) Responses of the gas sensor with and without Au decoration at room temperature. The inset shows a linear fit for the response of the gas sensor based on Au-decorated Ni-doped ZnS nanospheres at a low HCHO gas concentration.

Before testing, target gases with different formaldehyde concentrations are needed. We injected certain volumes of formaldehyde, ethanol, acetone, toluene, and ammonia solutions into a 1000 mL empty glass bottle with fresh air, to prepare target gases of various concentrations. For the preparation of ultra-low-concentration formaldehyde gas, two glass bottles with valves of the same capacity were used to prepare formaldehyde standard samples of four different concentrations by the double dilution method. In brief, one bottle contains a certain amount of gas. A vacuum pump is used to vacuum the other glass bottle. Then, two bottles are linked together to divide the gas into two halves. Finally, 2 bottles of the target gas were injected into pure air to achieve atmospheric pressure. Figure 3a shows the dynamic responses of gas sensors consisting of Ni-doped ZnS nanospheres and Au-decorated Ni-doped ZnS nanospheres to reducing formaldehyde gas at concentrations in the range of 1–10 ppm at room temperature. Figure 3b,c shows enlarged images of parts of the response curves of Ni-doped ZnS nanospheres and Au-decorated Ni-doped ZnS nanospheres at room temperature, respectively, corresponding to 5 ppm formaldehyde in Figure 3a, to show the moments of gas sensor introduction and extraction of the target gas. Both the sensors were exposed to 0.625, 0.125, 0.25, 0.5, 1, 2, 5, and 10 ppm formaldehyde gas to examine their gas-sensing responses. As the sensors were exposed to the target gas, the electric current increased immediately until it nearly reached a plateau. When the target gas was evacuated from the sensor, the electric current decreased significantly to its initial value in the presence of pure air. The gas sensor without Au decoration showed responses of 2.0–6.67 to 0.25–10 ppm formaldehyde at room temperature. By contrast, the Au-decorated gas sensor showed responses of 2.3–14.97 to 0.25–10 ppm formaldehyde at room temperature. Even if the formaldehyde concentration is as low as 62.5 ppb, the current signal of the Au-decorated gas sensor will change to 30%, while that of the gas sensor without Au decoration is insufficient. The results indicate that Au decoration of the Ni-doped ZnS nanospheres significantly improved the performance of the gas sensor. In Figure 3b,c, the response time of the Au-decorated gas sensor is 29 s, and that of the gas sensor without Au decoration is 34 s. Both the gas sensors have a recovery time of 2 s. However, we found that, when the target gas was evacuated, the current value of the Au-decorated gas sensor was closer to the baseline. Although both of the gas sensors respond very quickly, the Au-modified sensor shows a faster response and greater recovery speed. Figure 3d plots the relationship between response and formaldehyde concentration for the two gas sensors. The response value of the gas sensor increases with the increase of formaldehyde concentration. The curve of the Au-decorated Ni-doped ZnS gas sensor is linear at low concentrations, and the sensitivity reached 5.24. 

The theoretical detection limit (DL) or limit of detection (LOD) is very important for sensor application and can be defined as the lowest quantity of a substance that can be distinguished from the absence of that substance. In practical application, the detection limit can be estimated by the method reported in previous works [28,29,30]:

DL = (k × S_B_)/sensitivity, (1)

where S_B_ is the standard deviation; k is the confidence factor, which is taken as 3 in general; and sensitivity is the slope of the linear calibration graph of low concentration of the analyte. Thus, the DL can be estimated to be approximately 67.5 ppb for the Au-decorated Ni-doped ZnS gas sensor. However, for the sensor without gold decoration, there was almost no response at a concentration of 62.5 ppb. Both the sensors have rapid response and recovery (<34 s and ~2 s, respectively). This result clearly indicates that transition metal (Ni) doping is an effective strategy to shorten the sensor response time, as well as enhance response to formaldehyde (HCHO). Furthermore, the transition metal doping may reduce the working temperature of the sensor to allow detection at room temperature. This is important for ZnS gas sensors because ZnS tends to be oxidized at high temperatures [31]. This room temperature sensor will have more stable sensing characteristics. 

**Figure 4:**
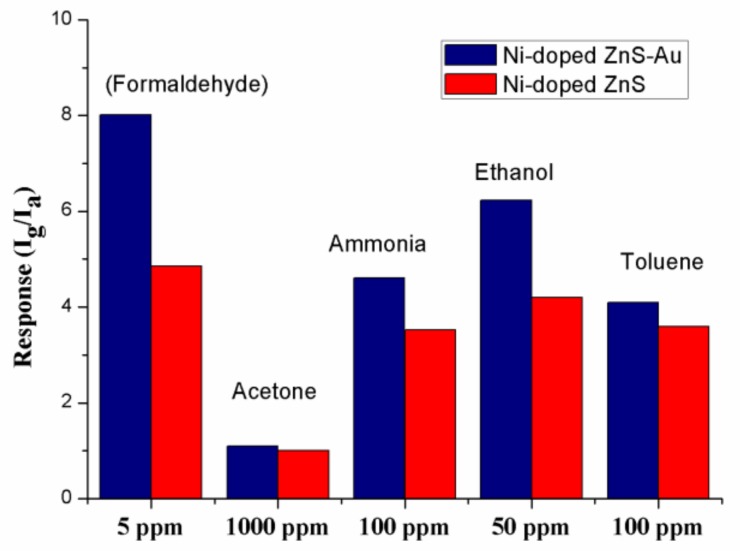
Sensitivities of the gas sensors based on pristine ZnS nanospheres and Au-decorated Ni-doped ZnS nanospheres to different gases at room temperature.

It is widely accepted that excellent selectivity is one of the essential characteristics of a high-performance gas sensor [7]. Figure 4 shows histograms of the sensors based on Au-decorated Ni-doped ZnS nanospheres and Ni-doped ZnS nanospheres toward various volatile organic compounds (VOCs). All the gases were tested at room temperature. Clearly, the gas sensor shows superior sensitivity towards formaldehyde compared to other gases. Based on this result, we may conclude that the selectivity of Au-decorated Ni-doped ZnS nanospheres toward formaldehyde gas is excellent. 

The response values to different gases are different at room temperature, which may be caused by the characteristics of materials. Previous studies have demonstrated that the energies of adsorption, desorption, and reaction on materials are different for different gases; therefore, the response values are different at the same operating temperature [32,33]. 

**Figure 5:**
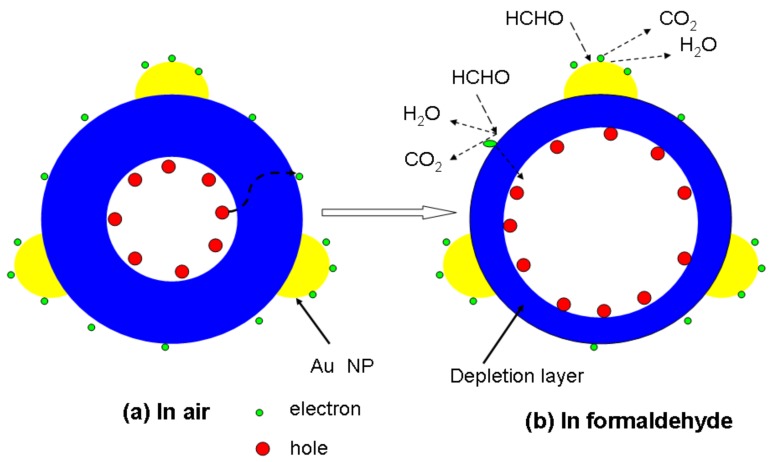
Schematic images of Au nanoparticle-decorated Ni-doped ZnS nanosphere when it is exposed to (**a**) air and (**b**) formaldehyde.

The gas sensor with Au nanoparticle decoration showed enhanced formaldehyde gas-sensing properties compared to that without Au decoration. Here, we suggest a possible mechanism for spherical nanostructures, as shown in Figure 5. The transportation of charge during the adsorption/desorption of gas species on the surface of the sensing structure is ascribed as response and recovery times of the target gas. In this sensing structure, the response and recovery times for formaldehyde gas were 29 s and 2 s, respectively. The fast response can be attributed to the following factors. First, catalytic and spillover effects between Au nanoparticles and Ni-doped ZnS nanospheres expedite the response and recovery reaction. In air, oxygen molecules can be adsorbed on the surface of the ZnS nanospheres to form chemisorbed oxygen (O^−^, O^2−^) by capturing free electrons from the conduction band as in the following reactions [34,35]:

O_2_(gas) → O_2_(ads) (2)

O_2_(ads) + e^−^ → O_2_^−^(ads) (3)

O_2_^−^(ads) + e^−^ → 2O^−^(ads) (4)

O^−^(ads) + e^−^ → O^2−^(ads) (5)

A depletion layer is formed in the surface region of nanospheres because of the consumption of electrons in the surface region of ZnS nanospheres, which results in an increased electrical resistance of the nanospheres. When the ZnS sensor is exposed to reductive VOCs such as formaldehyde, the target gas molecules can react with the chemisorbed oxygen and release the trapped electrons back to the conduction band of ZnS nanospheres, which increases the free-electron concentration and decreases the resistance of the sensor. Therefore, if a catalyst exists, the reaction will be more intense, resulting in an improved response of the sensor. Furthermore, as illustrated in Figure 5, owing to the catalytic effect of Au nanoparticles [31,36], more chemisorbed oxygen will be adsorbed on Au nanoparticles. The spillover effect of Au nanoparticles [37,38,39] enables the chemisorbed oxygen species to be easily transported and distributed on the surface of the nanospheres, and expedites the reactions at the interface between Au nanoparticles and ZnS nanospheres. 

Second, the local charge reservoir layer resulting from transition metal doping reduces the charge-transfer distance and response time. It is well known that the sensing function of nanomaterials is dominated by the surface resistance, which is regulated by the depletion layer, rather than the core area. Without Ni doping, the ZnS nanospheres have a homogeneous distribution of charge in the entire core area. However, the holes aggregate at the outer side of the core part. Thus, the transfer distance of the charge from the core to surface defects will be greatly shortened compared to the non-doped counterpart. Therefore, when ZnS nanoparticles are exposed to air, electrons are attracted to adsorbed oxygen species from the body. Thus, the depletion layer of the nanoparticles would enlarge, increasing the electrical resistance. On the other hand, when the nanoparticles are exposed to reductive gas molecules such as formaldehyde, as shown in Figure 5b, formaldehyde gas reacts with oxygen ions. This reduces the width of the depletion layer as well as the resistance of the sensor. 

As we know, chemical reactions occur on the nanostructure surface. Therefore, the area of the electron depletion layer in the entire crystal is an important factor [40]. Decreasing the crystal size and making the surface rougher will increase the proportion of the electron depletion layer in the crystal. Thus, more free electrons get released upon exposure to formaldehyde gas in the Au-decorated sensor. Consequently, the resistance of the sensor will decrease significantly, and the sensor will show a higher response. Owing to the above reasons, the gas sensor consisting of Au nanoparticle-decorated Ni-doped ZnS nanospheres showed an improved gas-sensing response. 

## 4. Conclusions

In summary, Au nanoparticle-decorated Ni-doped ZnS nanospheres were prepared for use as gas sensors by using a liquid phase reaction followed by the vacuum evaporation of Au and thermal annealing. The gas sensor based on Au nanoparticle-decorated Ni-doped ZnS nanospheres showed responses of 2.3–14.97 in a formaldehyde concentration range of 0.25–10 ppm at room temperature, and the detection limit can reach 67.5 ppb. The responses were superior to those obtained by the gas sensor based on Ni-doped ZnS nanospheres without Au decoration. Furthermore, the sensor with Au nanoparticle decoration showed a faster response. The significant improvement in performance of the sensor is attributed to the quantity of singly ionized oxygen vacancies. Furthermore, the enhanced sensor response by Au functionalization is mainly due to the increase in electrons from the localized surface plasmons of the Au nanoparticles to the conduction band of ZnS, as well as the combination of spillover effects and the enhancement of chemisorption and dissociation of gas.
